# Pharmacogenetics of FSH Action in the Male

**DOI:** 10.3389/fendo.2019.00047

**Published:** 2019-02-28

**Authors:** Maria Schubert, Lina Pérez Lanuza, Jörg Gromoll

**Affiliations:** ^1^Department of Clinical and Surgical Andrology, Centre of Reproductive Medicine and Andrology, University Hospital Münster, Münster, Germany; ^2^Centre of Reproductive Medicine and Andrology, University Hospital Münster, Münster, Germany

**Keywords:** idiopathic male infertility, FSH, spermatogenesis, genetics, single nucleotide polymorphism (SNP), pharmacogenetic studies

## Abstract

Male infertility is a major contributor to couple infertility, however in most cases it remains “idiopathic” and putative treatment regimens are lacking. This leads to a scenario in which intra-cytoplasmic spermatozoa injection (ICSI) is widely used in idiopathic male infertility, though the treatment burden is high for the couple and it entails considerable costs and risks. Given the crucial role of the Follicle-stimulating hormone (FSH) for spermatogenesis, FSH has been used empirically to improve semen parameters, but the response to FSH varied strongly among treated infertile men. Single nucleotide polymorphisms (SNPs) within FSH ligand/receptor genes (*FSHB/FSHR*), significantly influencing reproductive parameters in men, represent promising candidates to serve as pharmacogenetic markers to improve prediction of response to FSH. Consequently, several FSH-based pharmacogenetic studies have been conducted within the last years with unfortunately wide divergence concerning selection criteria, treatment and primary endpoints. In this review we therefore outline the current knowledge on single nucleotide polymorphisms (SNPs) in the FSH and FSH receptor genes and their putative functional effects. We compile and critically assess the previously performed pharmacogenetic studies in the male and propose a putative strategy that might allow identifying patients who could benefit from FSH treatment.

## Background

**Figure 1:**
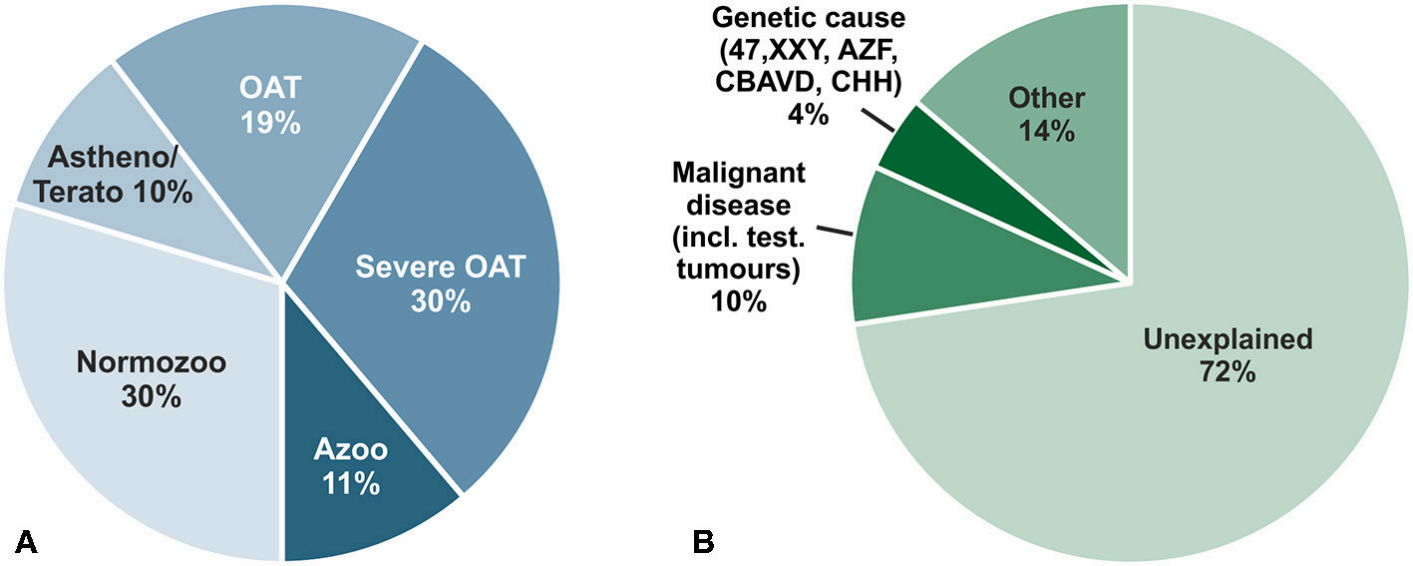
**(A)** Descriptive diagnoses according to semen analyses of 26,091 men in infertile couples who attended the Center of Reproductive Medicine and Andrology (CeRA), Münster over the last 30 years. **(B)** Clinical diagnoses in the same men. Data from Androbase^©^, the clinical patient database. Adopted from Tüttelmann et al. (2).

Infertility concerns at least 15% of couples in western countries in their reproductive age and in 50% of all cases male factor infertility contributes essentially (1). Several factors such as genetic or oncological causes (e.g., testicular tumors) clearly contribute to impaired spermatogenesis; but a specific cause can only be attributed to 28% of unselected infertile men. This leaves around 72% of men with idiopathic/unexplained infertility or with minor causes, e.g., low grade varicocele, not sufficient to explain their underlying infertility (2) (Figure 1). Reduced spermatogenesis is also mirrored in a large fraction of about 60% of infertile men by increased Follicle-stimulating hormone (FSH) and decreased Inhibin levels due to a disturbed feedback loop within the hypothalamic-pituitary–testis axis. Men displaying this endocrine pattern also exhibit reduced testicular volume, decreased serum Testosterone and increased Luteinizing hormone (LH) levels as a general sign for a hypergonadotropic hypogonadism (2). In a small fraction of men, however, this feedback loop is differentially regulated and characterized by lowered testicular volume, reduced sperm count but subnormal to normal FSH levels for so far unknown reasons. This group of idiopathic infertile men could resemble a target group for which a FSH treatment could be beneficial (3). By increasing FSH serum levels spermatogenesis could be stimulated further, a scenario not valid for the group of hypergonadotropic hypogonadal patients who already have elevated FSH serum levels.

Although the essential role of FSH for spermatogenesis has been recognized for decades (6) and several studies on FSH treatment for infertile men have been conducted, the overall outcome is disappointing. The only significant improvement which could be deduced from the different FSH studies is improved pregnancy rates (7, 8). However, clinical consequences cannot be drawn from these results. According to the recent EAA guidelines, FSH treatment can be offered in selected men [normogonadotropic with idiopathic oligo- or oligo-astheno-teratozoospermia (OAT)], however, with low evidence for success only (9). The Italian Society of Andrology and Sexual Medicine recently suggested in a consensus statement to use FSH to increase sperm concentration and motility in infertile normogonadotropic men with idiopathic oligozoospermia or OAT, with moderate evidence grading. The treatment with FSH is suggested in these men to improve both spontaneous pregnancy as well as pregnancy rates after ART (10). To which extent these recommendations can be adapted by other European countries remains to be seen.

Nowadays in clinical routine, if no causative factor for impaired infertility can be identified and treated, the agreed on procedure for men with idiopathic infertility is to undergo assisted reproduction (ART), which is mainly due to the fact that a clear treatment option cannot be offered or does just not exist. Recent data from the German *in vitro* fertilization (IVF) registry indicates that there is an increasing rate for not only the usage of ART but also for replacing IVF by intracytoplasmic sperm injection (ICSI) treatment (11). This strong tendency can also be observed worldwide, despite the fact that assisted reproduction techniques and treatment is putting the burden on the female side only. Besides the risks for women undergoing ART e.g., ovarian hyperstimulation syndrome, complications by oocyte retrieval and re-implantation, there is also clear evidence that progeny health might be affected by a treatment such as ICSI. In the current literature putative risks of ART for congenital malformations, epigenetic disorders, chromosomal abnormalities, subfertility, cancer and impaired cardio-metabolic profiles are discussed (12). These potential risks may be due to the fact that a routine ICSI procedure circumvents nearly all barriers naturally existing for fertilization such as sperm selection, competition etc.

Taken together, the clear tendency in reproductive medicine to neglect male infertility as a treatable condition and instead to routinely apply ART, demands novel strategies for curing male infertility. The currently most promising approach is to induce full spermatogenesis by FSH treatment. However, it is also clear that FSH treatment is not beneficial for all subfertile/infertile men, and that a personalized treatment regimen which takes into account clinical and genetic factors controlling spermatogenesis might resemble the most promising approach.

In this review we therefore outline the current knowledge on single nucleotide polymorphisms (SNPs) in the FSH beta and FSH receptor genes, we compile and critically assess the previously performed pharmacogenetic studies in the male and propose a putative selection strategy that might allow identifying patients who could benefit from FSH treatment. Detailed information on pharmacogenetics are comprised in Box 1.

Box 1 **Pharmacogenetics**

The general principle of a pharmacogenetics approach is to optimize drug efficacy, minimize toxic effects based on the inherited genetic variation in each individual. In general genetic variants such as single nucleotide polymorphisms (SNPs) with a frequent prevalence in the population are being used (Minor allele frequency >5%), otherwise the applicability for pharmacogenetic approaches will be limited to individual persons only. For further details on nomenclature of variants, [see (4)].

This personalized medical approach has the potential to identify the most appropriate patient for which a given treatment is really beneficial. Pitfalls of personalized approaches based on genetic information are to which extent variability may be attributed to biological factors (e.g., general health, age etc.) and environmental/behavioral factors (e.g., smoking) giving rise to responders and non-responders. Caution has to be given to the fact that the clinical outcome of a pharmacogenetic study might vary due to either genetically distinct populations or that a subset of unfavorable genetic variants might interfere with the variant being tested and thus bias the expected drug-gene/outcome interaction (5).

### Follicle-Stimulating Hormone Action in Sertoli Cells

For qualitative and quantitative normal spermatogenesis an intact hypothalamic-pituitary-gonadal (HPG) axis is essential. Gonadotropin releasing hormone (GnRH) is released by the hypothalamus. In turn, GnRH stimulates the pituitary to secrete LH and FSH. LH stimulates production of testosterone in Leydig cells, which negatively feeds back to the pituitary as well as the hypothalamus in order to modulate the production of GnRH and by this gonadotropin levels (13). FSH synthesis and secretion depends on slow GnRH pulses (every 2–4 h), while rapid GnRH pulses (every 30 min) lead to preferential secretion of LH (14).

During the “mini puberty” (12–18 months of age) the HPG axis is activated for normal genital development, later on during development the HPG axis is again activated with the onset of puberty. During prenatal and prepubertal stage, FSH stimulates Sertoli cell proliferation and by this determines their final number and subsequently testicular size. The proliferation and functional maturation of Sertoli cells is controlled and terminated by thyroid-stimulating hormone (TSH) (15).

Sertoli cells (SCs) are part of the seminiferous tubules of the testes and play a key role in spermatogenesis. They are “nurse cells” as they provide nutritional support for germ cell development. Moreover, they contribute to the spermatogonial stem cell (SSC) niche and are this way indispensable for functional spermatogenesis (16). Sertoli cells and their metabolism are regulated by hormones (17). Some hormone receptors are solely expressed in Sertoli cells which underlines the importance of hormonal signaling for spermatogenesis (17). Thus, Sertoli cells transduce endocrine signals into the paracrine regulation of germ cells (16).

Busch et al. showed that boys with the genotype *FSHB* c.-211GT/TT and *FSHR* c.-29AA entered puberty later, which indicates that the overall endocrine network as well as FSH action might be affected in early phases by SNPs (18). In the adult stage, proliferation is ceased in mature Sertoli cell and FSH stimulates the proliferation of spermatogonia (19). In humans FSH mainly regulates sperm output of the seminiferous epithelium by controlling the expansion of premeiotic germ cells (20). FSH also influences the proliferation of type A spermatogonia upregulating nerve growth factor inducible gene B (NGFI-B, also known as Nur77) which increases the expression of glial cell line-derived neurotrophic factor (GDNF) in SCs (21). GDNF in turn supports the proliferation of germinal stem cells (GSCs) and other undifferentiated spermatogonia (22).

### Follicle-Stimulating Hormone Signal Transduction

The FSH receptor (FSHR) belongs to the 7 transmembrane domains receptor (7TMR) family of G-protein coupled receptors and is only expressed in Sertoli cells (23). FSH binding induces a conformational change of the FSHR especially within the TM domains 5 and 6 which cause intracellularly the dissociation of α- and βγ- subunits of G protein heterotrimer inside the cell. Subsequently, the α-subunit binds to and triggers adenylyl cyclase, which leads to an increase of cAMP levels (24). The main signal transduction pathway for the FSHR is the cAMP-PKA pathway. Its activation leads to a release of the catalytic subunit of protein kinase A (PKA); followed by phosphorylation of enzymes and proteins. Moreover, it targets the cAMP response element binding protein (CREB) which activates transcription of FSH-dependent genes (25). The MAP kinase cascade and extracellular-signal regulated kinase (ERK) get activated most likely via cAMP interactions with guanine nucleotide exchange factors (GEFs) and activation of Ras-like G proteins. By GEFs the phosphatidylinositol 3-kinase (PI3-K) pathway gets activated which leads to an activation of protein kinase B (PBK) (26). The PI3-K pathway plays an important role as it regulates several biological processes e.g., glucose uptake, oxidative burst and mitogenesis (17). Moreover, FSH causes an increase in intracellular calcium mediated by cAMP. Elevated calcium concentrations cause an activation of calmodulin and CaM kinases which result in downstream effects including the phosphorylation of CREB. FSH inducts phospholipase A2 (PLA2) and the release of arachidonic acid (AA) and the activation of eicosanoids (26). These different pathways activate different transcription factors thereby stimulating the transcription of FSH-targeted genes (27). Consequently, Sertoli cells transduce signals from FSH into production of necessary factors for germ cell nutrition and differentiation.

## Genetics of Follicle-Stimulating Hormone/Follicle-Stimulating Hormone Receptor

### The Follicle-Stimulating Hormone Beta (FSHB) Subunit Gene

**Figure 2:**
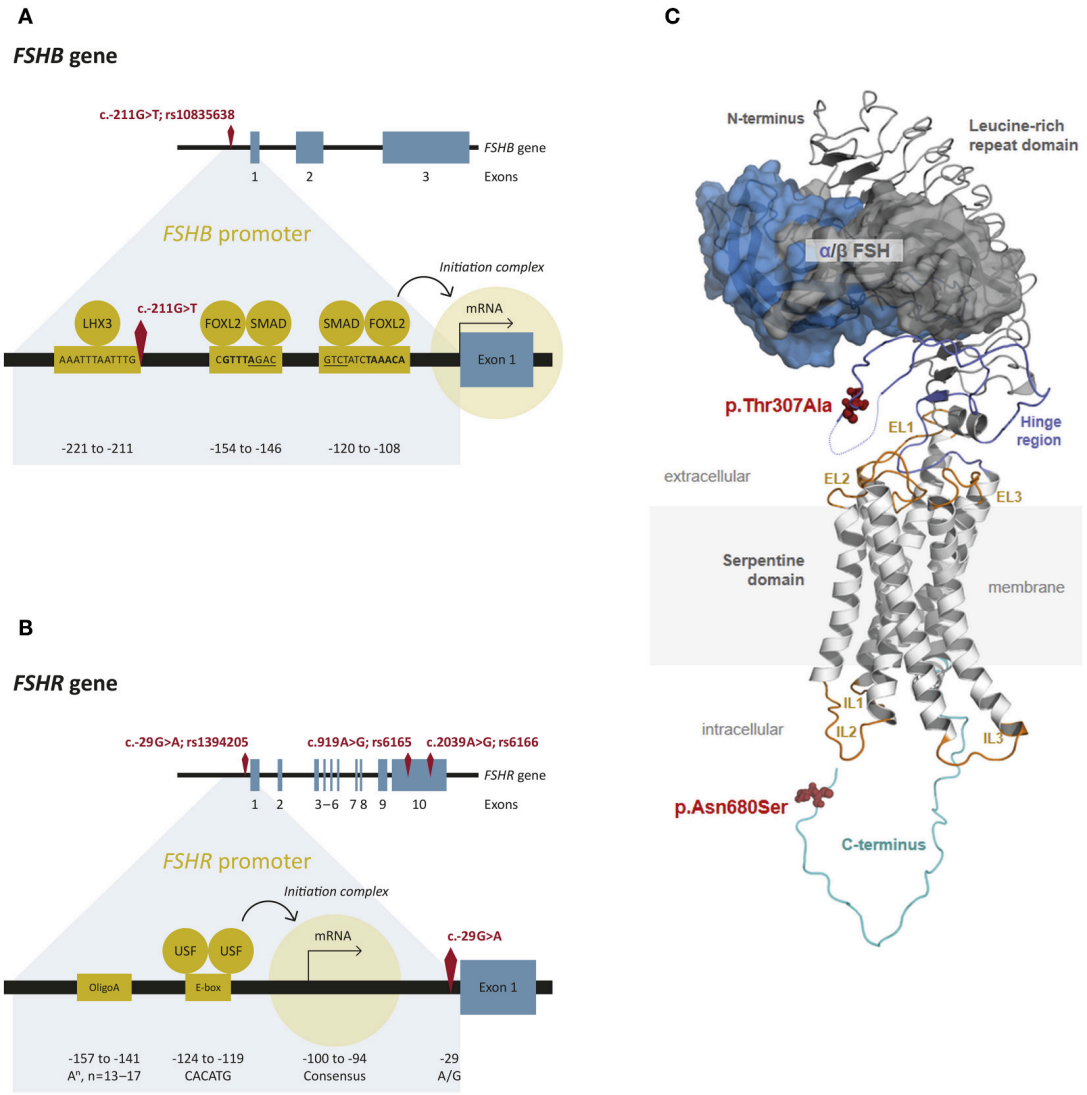
FSHB and FSHR: Gene, promotor and protein structure. **(A)** Structure of the *FSHB* gene and promotor. The *FSHB* gene consists of three exons. The transcription factor LHX3 binds to the *FSHB* promotor as well as FOXL2 (binding sites are bold) and SMAD (binding sites are underlined) (28). The transcription start site is located on exon 1. The SNP rs10835638 (c.-211G>T) is located in the promotor region of the *FSHB* gene. **(B)** Structure of the *FSHR* gene and promotor. The gene consists of 10 exons. The transcription factors USF bind to the E-box and the transcription starts. The SNP rs1294205 is located in the promotor region (c.-29G>A) of the *FSHR* gene. The SNPs rs6165 (c.919A>G) and rs616 (c.2039A>G) are located in exon 10. **(C)** Protein structure of FSH and FSHR. A three-dimensional homology model of the FSH/FSHR complex is shown. The 7 TMD, constituted by transmembrane helices connected by intracellular (IL) and extracellular (EL) loops, was modeled based on the determined active structure-conformation of the β2-adrenergic receptor (29). The (monomeric) extracellular complex between the hinge region, the leucine-rich repeat domain, and FSH were taken as suggested by a structure determined for a fragment (24). The hinge region structurally links the leucine-rich repeat domain with the 7 TMD. The FSHR (backbone white-7 TMD, light blue-hinge, light gray leucine-rich repeat domain) binds the hormone [FSHβ (dark gray) and FSHα (blue), surface representation] at the extracellular side between the leucine-rich repeat domain and the hinge region. The exact orientation between the different components to each other is still unclear. The p.Thr307Al variant is located in the hinge region, where a derived structure is not known yet. The intracellular coiled loop (light green), where also not structural motifs are known yet, harbors the second amino acid variant p.Asn680Ser. The 3-D model of the FSH/FSHR-complex was kindly provided by Gunnar Kleinau (Charité Berlin, Germany).

FSH is a pituitary derived heterodimeric glycoprotein which consists of an alpha-subunit and a unique beta-subunit that determines biological specificity and provides specificity for receptor binding (14). The human *FSHB* gene (National Center for Biotechnology Information (NCBI) database https://www.ncbi.nlm.nih.gov/gene/2488; GeneID:2488; Locus tag:HGNC:3964) is located on chromosome 11p13 and consist of 3 exons (Figure 2A) (30). It encodes the FSH beta-subunit consisting of an 18-amino acid (aa) signal peptide and the 111-aa mature protein (31). The NCBI SNP database (https://www.ncbi.nlm.nih.gov/SNP/) lists 1380 SNPs in the gene region of *FSHB*, 114 SNPs in coding regions. Only very few SNPs have proven clinical relevance. One of them, SNP rs10835638 (c.-211G>T) is located in the 5′untranslated region within an evolutionary conserved element of the *FSHB* promotor (Table 1; Figure 2A), which leads to an influence on gene transcription (33). This SNP impairs LHX3 binding and induction of *FSHB* transcription. Thus, the SNP rs10835638 reveals significant functional importance (33). The regulation of *FSHB* transcription in gonadotropic cells is essential as the amount of the beta-subunit being transcribed is the rate limiting step in FSHB synthesis and defines how much mature hormone will eventually be secreted, as the alpha subunit is shared with LH, Thyroid-stimulating hormone (TSH) and human Chorionic Gonadotropin (hCG), and produced in excess. Hence, *FSHB* transcription is directly associated with its translation, secretion and serum levels (14).

**Table 1:** Minor allele frequencies of the most relevant SNPs within the *FSHB* and *FSHR* genes. Taken from the 1000 Genomes project (32).

**Gene**	**SNP ID**	**DNA nucleotide**	**Protein**	**Minor allele frequency (32)**
*FSHB*	rs10835638	c.-211G>T	Promoter, non-coding	T = 0.0839
*FSHR*	rs1394205	c.-29G>A	Promoter, non-coding	T = 0.3450
*FSHR*	rs6165	c.919A>G	p.Thr307Ala	T = 0.4922
*FSHR*	rs6166	c.2039A>G	p.Asn680Ser	C = 0.4073

### The Follicle-Stimulating Hormone Receptor (FSHR) Gene

The 76 kDa FSH receptor (FSHR) is a G protein-coupled receptor, which belongs to the rhodopsin-like receptor subfamily, and consists of 695 amino acids (34). The *FSHR* gene (NCBI database https://www.ncbi.nlm.nih.gov/gene/2492; GeneID:2492; Locus tag:HGNC:3969) is located on chromosome 2 p21-p16 and consists of 10 exons of which the first nine exons encode the extracellular amino-terminal domain of the receptor (34, 35). Exon 10 encodes the transmembrane and intracellular portions of the protein (Figure 2B) (36). Two human isoforms are known, one containing all exons (NM_000145) and the second one lacking exon 6 (NM_181446) (13). The extracellular domain contains a stretch of leucine-rich repeats essential for FSH binding and is encoded by exon 2-9 (Figure 2C) (36). The NCBI SNP database (https://www.ncbi.nlm.nih.gov/SNP/) lists 51.677 SNPs in the gene region of *FSHR*, 779 SNPs are located in coding regions. The following SNPs: rs6166 (c.2039A > G, p.N680S) and rs6165 (c.919A > G, p.T307A) (Table 1), which are located in exon 10 have been analyzed more thoroughly and are in linkage disequilibrium (LD). The SNP c.919A>G results in an amino acid exchange removing a potential O-linked glycosylation site in the hinge region of the receptor, the SNP c.2039A>G also results in an amino acid exchange causing a potential phosphorylation site in the intracellular domain of the receptor (37, 38). Other known SNPs rs1394205 (c.-29G>A) and rs115357990 [c.-114T>C; MAF 0.0126 (1000 Genomes Project)] are located in the promotor region of the *FSHR* gene (39).

### The Impact of *FSHB/FSHR* SNPs on Endocrine Function and Spermatogenesis

Interestingly, data on the putative impact of the several SNPs within the *FSHB* and *FSHR* genes have been mainly and firstly obtained from clinical studies and only later in part paralleled by experimental studies. There are only few *in vitro* studies available concerning the effect of *FSHB* and *FSHR* SNPs on FSH action in the male. This is mainly due to the fact that appropriate read-out systems for studying FSH function and corresponding SNPs functions do not exist. There are neither human gonadotropic nor Sertoli cell lines available. While it is generally believed that mouse gonadotropic cell lines such as the LßT2 cell line are useful and informative for the human as well, the situation for Sertoli cells is worse. The commercial available “Sertoli” cell lines are closer to peritubular cells than to Sertoli cells and therefore of only limited usefulness. Moreover, immortalized Sertoli cells tend to lose their intrinsic FSH receptor expression, making it difficult to study FSH action (40). Some groups have therefore stably transfected Sertoli cell lines with the FSH receptor to regain FSH sensitivity (41). The characteristic feature of losing FSH receptor during immortalization of testicular somatic cells can also be observed for the female pendant of Sertoli cells, the granulosa cells. One of the reasons for the shut-down of FSH receptor expression might be aberrant methylation of regulatory elements controlling receptor expression (42).

Usage of primary cells as a substitute for lacking immortalized cell lines is at least for adult human Sertoli cells also not an option, since there is currently no protocol available which allows to isolate intact Sertoli cells in sufficient amounts. This is mainly due to the fact that the Sertoli germ cell niche is so tightly interlinked, that most cell separation protocols lead to the destruction of these cells. Thus, there is a great need for cell model systems which would allow studying the impact of SNPs and mutations in the male.

Nevertheless, in the mouse gonadotropic cell line (LßT2) it could be shown that the *FSHB* c.-211G>T polymorphism, located on the promoter region has an effect on the binding of the LHX3 homeodomain transcription factor, which leads to an impaired binding and this way to a 50% decrease in transcriptional promotor activity (33).

The SNP in the promotor region of *FSHR* c.-29G>A, currently used in a number of FSH studies, was analyzed in murine Sertoli cells SK11, but a significant effect could not be shown (39). Conflicting to this result, another group showed that the *FSHR* c.-29G>A decreases transcriptional promoter activity by 56% for the A allele *in vitro* in Chinese hamster ovary (CHO) cells (43). While these results await further confirmation by additional experiments, one should note that there are more SNPs allocated in the core promotor region of the *FSHR* gene such as a highly variable oligo-A stretch and a SNP at position at c.-114T>C. Therefore, a valid investigation on the impact of the c.-29G>A SNP should include the other polymorphic sites as well (39).

The impact of the SNPs in exon 10 in *FSHR* was analyzed *in vivo* showing that a decreased activity of the FSHR has a clinical implication for female infertility (44–46) and *in vitro* (38, 47): Nordhoff and colleagues analyzed the increase of cAMP and Estradiol after FSH stimulation between the variants NN and SS in human granulosa cells and could not show a difference in the mentioned parameters (47). Simoni and Casarini also used human granulosa-lutein cells (hGLC), but analyzed the kinetics of different signal transduction pathways. The p.Asn680Ser FSHR variant led to decreased ERK1/2 activation and the FSHR seemed to be less active (38). Again, no data on the impact of these SNPs on Sertoli cells are available. Hence, there are some *in vitro* studies demonstrating the effect of SNP in *FSHB*/*FSHR* granulosa cells/cell lines, but more *in vitro* studies targeting the molecular impact of the SNPs on FSH signaling using Sertoli cells are needed.

### Clinical Impact of *FSHB/FSHR* SNPs on Spermatogenesis

The essential role of FSH for spermatogenesis is underlined by the identification and clinical characterization of inactivating *FSHB* mutations. Until now five men with such *FSHB* mutations have been described which all showed azoospermia and either very low or absence of FSH serum levels (31). Similar to this, several mutations and variants of the *FSHR* have already been described in humans (48–50). The first description of a human inactivating *FSHR* mutation (p.Ala189 Val) showed elevated FSH levels and abnormal sperm parameters, but no azoospermia in affected men (51). An activating *FSHR* mutation (p.Asp567Gly) was discovered by Gromoll et al. describing a hypophysectomized man who fathered children being under Testosterone treatment only (52). So far, there have been 11 inactivating - and 7 activating mutations of the *FSHR* described in men and women (53).

Along the findings of completely abolishing FSH action by inactivating mutation, minor genetic changes such as SNPs affecting *FSHB* transcription, FSH binding properties or FSH receptor sensitivity could impact male fertility too. In the *FSHB* gene, the SNP c.-211G>T (rs10835638) has a major effect on serum FSH concentration in men (30). This effect as well as reduced testis size, reduced sperm concentration and lower serum Inhibin B and Testosterone was also shown in a cohort of Italian (54), Baltic (55), and German men (56).

**Figure 3:**
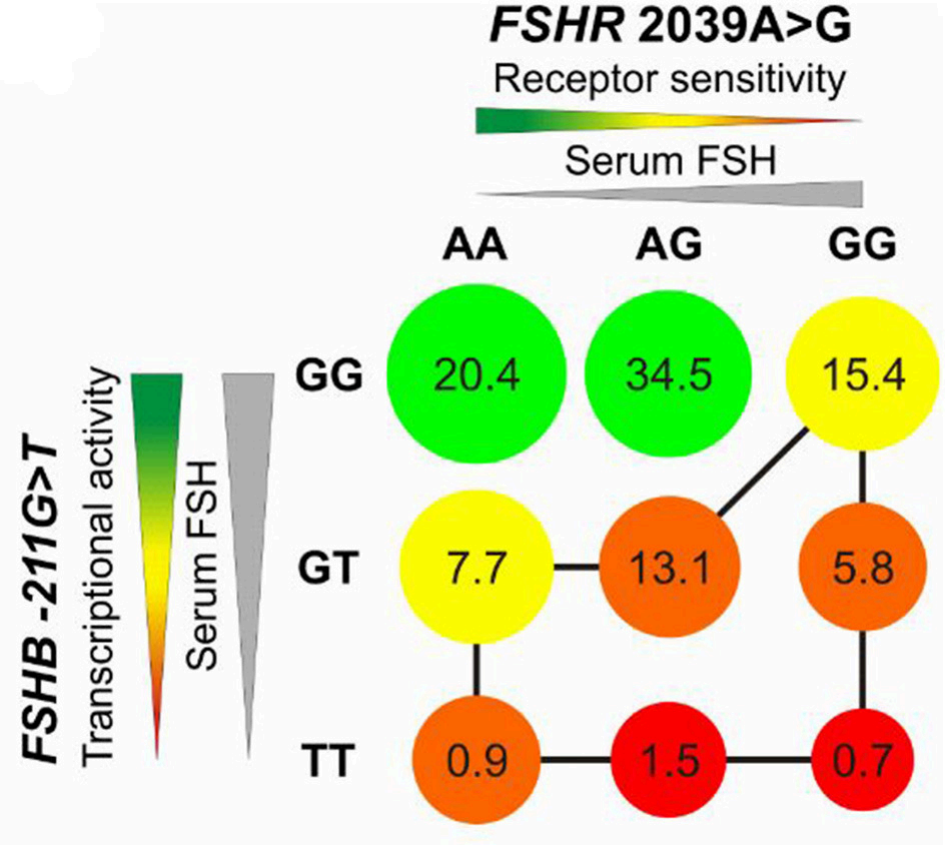
Impact of rs10835638 (*FSHB* c.-211G>T) and rs6166 (*FSHR* c.2039 A>G) on serum FSH, transcriptional activity of *FSHB* and receptor sensitivity of FSHR. Decreasing receptor sensitivity and transcriptional activity of *FSHB* lead to reduced testicular volume shown by circle diameter. The red color indicates unfavorable genotype, the green color a favorable genotype for reproductive fitness. The numbers show the percentage of carriers of combined genotypes in a German population group. The least favorable genotypes are marked with a black line. Men with TT/GG are predicted to show lowest testicular volume. Adopted from Tüttelmann et al. (56).

Tüttelmann et al. analyzed the combined effect of a SNP in *FSHB* and *FSHR* on male reproductive parameters. They found a marked dominant effect of *FSHB* c.-211G>T in combination with *FSHR* c.2039A>G on serum FSH and testicular volume. The T allele carriers of the SNP c.-211G>T showed reduced FSH, increased LH, lower testicular volume, lower sperm count and concentrations in comparison to GG homozygotes men (Figure 3) (56). A recent study on the impact of *FSHB* c.-211G>T in Italian men from Tamburino et al. described decreased FSH, LH, Testosterone, sperm count and testicular volumes in men with GT or TT in comparison to GG (57). Additionally, two population-based studies analyzed the effect of *FSHR* c.-29G>A in a Baltic and an Italian men cohort, respectively. Grigorova et al. showed an association between this SNP and FSH levels and Tamburino et al. showed that the *FSHR* c.-29G>A SNP is associated with higher FSH and LH in normozoospermic men. This effect could only be observed in normozoospermic men but not in men with alterations in conventional sperm parameters (58, 59).

The impact of the total phenotypic variance (SNP) was evaluated for the first time in a cohort of young Baltic men by Grigorova et al. (58). The *FSHB* –c.211G>T in combination with the *FSHR* c.-29G>A and the *FSHR* c.2039A>G explained 2,3% of serum FSH variance in young men as well as 1,4% serum Inhibin B, 1% Testosterone and 1,1% total testes volume. Additionally, in a cohort of infertile Estonian men the SNP combination affected 2,3% of serum FSH variance, 2,6% serum Inhibin B and 2% total testes volume (58). In a recent meta-analysis Wu et al. investigated the effect of four SNPs (*FSHB* c.-211G>T, *FSHR* c.-29G>A, *FSHR* c.919A>G, *FSHR* c.2039A>G) on male infertility. It seems that the combination of three SNP genotypes of *FSHR (FSHR* c.-29G, c.919A, c.2039A) results in protection against male sterility than either one alone (60).

## Clinical Impact of FSH in Infertile Men

### Clinical Studies in Infertile Men Using FSH Current Status

In the past multiple clinical studies were carried out on idiopathic infertile men receiving FSH therapy to increase birth and pregnancy rates in the couple. However, results were conflicting. Therefore, a Cochrane Review and a recent meta-analysis were carried out to further elucidate these diverse results (7, 61).

Attia and colleagues analyzed 6 randomized controlled trials (RCTs) in which application of FSH was compared to placebo or no treatment at all. The main results comprise a significant increase in spontaneous pregnancy rate and in live birth rate in couples with FSH treatment in the male. The authors critically conclude that these results are promising, but since number of RCTs and participants is small and the evidence is low, no final clinical conclusions can be drawn (7).

In the most recent meta-analysis Santi et al. evaluated similar endpoints of FSH treatment outcome in idiopathic infertile men in 15 studies. The key finding of this analysis, supporting the prior Cochrane analysis, was the significant improvement in pregnancy rate in FSH-treated men (61). Additionally, an increased pregnancy rate was observed after ART when applying FSH. The pregnancies achieved were independent of FSH preparation and duration of therapy. Interestingly, in the sub-analysis considering the treatment response in terms of sperm parameters, a significant increase was only seen in sperm concentration, whereas other semen parameters did not change.

These are in principle promising results for FSH treatment of idiopathic infertile males, however the most susceptible parameters like FSH serum level or basal sperm count do not contribute to distinguish patients who will benefit from treatment from those who won't; predictive markers are therefore eagerly warranted (61) and a pharmacogenetic approach was suggested by several groups (3, 38).

### State of the Art of Pharmacogenetic FSH Studies in Men

To our knowledge, there are currently only 4 studies (54, 62–64), that chose a FSH-based pharmacogenetic approach (Table 2). Our literature search was conducted in MEDLINE (PubMed), the Cochrane Library, Scopus and UpToDate. Search was last updated in October 2018.

**Table 2:** Overview of the current FSH-based pharmacogenetic studies.

**Study**	**Study type**	**Study size**	**Female factor**	**SNP selection**	**Inclusion criteria**	**FSH**	**Prim. end-point**	**Pharmaco-genetic results**
Selice et al. (63)	Prospective RCT Single center	70/35	/	FSHR p.T307A p.N680S (**AS/AS**, **TN/AS**, **TN/TN**)	FSH 1-8IU/l, sperm conc.: < 20 × 10^6^/ml, testicular cytology: hypo-spermatogenesis	rFSH/ 150IU thrice weekly/3 months	TSC	**AS/AS**: ↑ **TN/AS**: ↑ **TN/TN**: –
Ferlin et al. (54)	Prospective Single center	67/0	no etiology for female infertility	*FSHB* c.-211G>T **(GG, GT, TT)**	FSH ≤ 8IU/l TSC < 40 × 10^6^Mill/ejac. (Azoospermia incl.)	rFSH/ 150IU thrice weekly/ 3 months	TSC	**GG, GT, TT**: ↑ (TT most impressive)
Simoni et al. (64)	Prospective multicenter, longitudinal, open-label, two-arms	55/0	no etiology for female infertility	FSHR p.N680S **(S/S, N/N)** *FSHB* c.-211G>T **(GG, GT/TT)**	FSH < 8IU/l DFI >15% (Oligo- Normo-zoospermia)	rFSH/ 150IU every 2nd day/ 3 months	DFI	FSHR p.N680S **N/N**: ↓ FSHR p.N680S **N/N** and *FSHB* c.-211G>T **GG**: ↓
Casamonti et al. (62)	Prospective, single center	40/0	/	*FSHB* c.-211G>T (**GG**, **GT/TT**), FSHR p.N680S (**S/S**, **S/N** and **N/N**) c.-29G>A (**GG**, **GA/AA**)	FSH < 8IU/l Oligo-or Astheno- or Terato-zoospermia, or OAT	hpFSH/ 75IU every 2nd day/3 months	sperm HBA	*FSHB* c.-211 **GG, GT/TT**: ↑ FSHR p.N680S **S/S**, **S/N** and **N/N**: ↑*FSHR* c.-29G>A **GG**, **GA/AA**: ↑

*First author of the study and respective study type is listed. The study size comprises total number of treated subjects/and total number of controls. If no female factor was described, this is indicated by a slash. The SNPs are given according to their nomenclature; the bold print indicates the respective alleles coding SNPs are indicated by their corresponding amino acids. Inclusion criteria are listing specific criteria only, for more general inclusion and exclusion criteria we refer to the original manuscripts. FSH treatment is pictured by type of FSH/dosage/ and duration of treatment. Only primary endpoints are listed, and the significantly obtained pharmacogenetic results refer to this parameter. The arrows indicate an increase or decrease of the primary endpoint parameter. For further results on secondary endpoints and insignificant results see respective studies*.

*RCT, randomized controlled trial; TSC, total sperm count; rFSH, recombinant FSH; hpFSH, highly purified FSH; DFI, DNA fragmentation index; sperm HBA, sperm hyaluronan binding assay*.

Records for: FSH/male infertility (*n* = 2245); FSH/male infertility/gene (*n* = 292); *FSHB* gene/male infertility/SNP (*n* = 23); FSH/male infertility/pharmacogenetics (*n* = 9). The four pharmacogenetics studies identified via the literature search are being discussed in terms of study design, selection criteria, treatment, endpoints and results.

#### Study Design

The four studies have in common a prospective approach, and were, except for the study by Simoni et al, carried out in a monocenter setting. The number of subjects treated with FSH was between *n* = 40–70. Only one study had a 2:1 randomization to a cohort of patients who did not receive treatment and was followed-up (63); all other studies only included subjects that received FSH treatment. No placebo controls were included in any of these studies. Statistical power analyses considering the primary end-point variation was provided by only one study (64).

#### Selection Criteria

The general selection criteria for the four studies were quite homogenous; more heterogeneity was evident in terms of specific inclusion criteria (see below and Table 2). All studies included male patients with idiopathic infertility, excluding major factors affecting spermatogenesis such as karyotype anomalies, Y-chromosomal microdeletions and congenital bilateral aplasia of the vas deferens. (See respective studies for further inclusion and exclusion criteria). FSH was within the regular range (< 8IU/l), this was also true for LH, Testosterone, Prolactin, Inhibin B and Estradiol. Heterogeneity exists among inclusion criteria for sperm parameters. This varied from patients with azoospermia or severe OAT, to patients with normozoospermia and “only” increased DNA fragmentation index (DFI). One group selected for hypospermatogenesis (reduced number of germ cells without maturation arrest, via fine needle aspiration) as inclusion criterion (see Table 2 for details). With respect to the genetic composition the *FSHB* promoter region (c.-211G>T) or the *FSHR* (c.919A>G, c.2039A>G, c.-29G>A) or a combination was chosen. Interestingly, the pharmacogenetic approach in 3 of the 4 studies was performed on *FSHR* SNPs, for which the clinical impact in men is still under debate. In several studies it was shown previously that the impact of the FSHR p.N680S polymorphism only slightly influences reproductive parameters (65–67). Surprisingly, only two studies reported on the female partners and comprised inclusion (regular ovulation and tubal function) and exclusion criteria (endometriosis, endocrine abnormalities (polycystic ovaries, anovulation, infections). Since female factors present a major factor for pregnancy rates, study results neglecting these parameters should be handled with caution.

#### FSH Treatment

Great heterogeneity exists among the FSH therapy concerning dosages and time of the application. In the respective studies treatment varied from 75 IU highly purified FSH (hpFSH) every other day to 150 IU recombinant FSH (rFSH) thrice weekly. The treatment period was 3 months in all studies, some accompanied by a follow-up (wash-out) period of further 3 months. In the most recent meta-analysis Santi et al. conclude that the positive effect of FSH (on spontaneous pregnancies and pregnancies after ART) was not dependent on the kind of FSH: hpFSH or rFSH (61). In the current EAA guidelines on the management of OAT treatment with “FSH can be suggested with low evidence in selected men with idiopathic oligozoospermia or OAT” (9). However, no further information on the dosages and the time of application is given.

#### Endpoints

The primary endpoints chosen were either sperm parameters like total sperm count (TSC), functional sperm tests like DFI or sperm-HBA (hyaluronic acid binding capacity). The latter being a biomarker for complete spermatogenesis, as suggested by the authors (62), since only mature spermatozoa, which have correctly completed spermiogenesis, express receptors for hyaluronic acid (68). DFI was chosen as endpoint, since it can be a predictor of the probability for conception. By using the TUNEL method, a distinction between viable cells (brighter DFI fraction) and dead cells (dimmer fraction of DFI) could be made (69, 70).

Considering the improvement of impaired fertility parameters in patients with idiopathic infertility by FSH as primary objective, one would rather see known and commonly accepted sperm parameters (i.e., TSC, motility) or pregnancy rate as directly linked parameters, rather than functional sperm tests. Especially tests such as DFI and HAB are interesting, but not reliable measures that are accepted as norms and/or standards in evaluation of fertility. These measures reveal further relevant information, but should therefore rather be considered as secondary endpoints. In the four studies the secondary endpoints were quite homogenous, reflecting hormonal parameters (i.e., FSH, LH, Testosterone), sperm parameters (TSC, sperm concentration, motility, morphology), clinical parameters (testicular volume) and pregnancy rate (Table 2).

#### Study Results

Selice et al. having the only study that included a control group (without treatment), showed significant increase in TSC, sperm concentration, motility and morphology in subjects with at least one serine in position 680, whereas patients homozygous for TN/TN showed no significant change in any semen parameters (Table 2) (63). However, the authors did not compare these parameters between the treated—and the untreated group. The only reference to the untreated group was to show that in these subjects the parameters did not change significantly upon follow-up. Evaluating polymorphisms in the *FSHR* as putative predictive factors for response to FSH treatment, a comparison between treatment and no treatment would have revealed additional valuable information.

The group around Casamonti et al. chose the sperm-HBA binding capacity, as biomarker for fully completed spermatogenesis, as primary endpoint. As secondary objective they stratified the patients according to the SNPs in *FSHB* c.-211G<T and *FSHR* c.2039A>G and c.-29G>A in order to find predictive markers for HBA responsiveness. Over all groups (irrespective of SNP) an increase of HBA-binding capacity was observed after short-term and 3 months of treatment, this increase was also evident in secondary parameters like TSC and total motile sperm count (TMSC) (62). Substratifications of the groups for sperm parameters, baseline-HBA, clinical parameters, pharmacogenetic parameters were carried out to identify predictive factors, which contribute to responsiveness of increased HBA (see Casamonti et al. for detailed results). In terms of the stratified SNP-groups, there was no clear-cut effect of the genotype of the SNP in predicting response to treatment, neither with regard to classical semen parameters, nor to HA binding capacity. However, the patient number of the study was probably too low to make such comparisons, since the study was statistically not powered for a pharmacogenetic approach. Additionally, the impact of sperm-HBA capacity needs to be discussed further in terms of clinical relevance for male infertility.

The pharmacogenetic approach by Ferlin was a subanalysis (*n* = 67) of a large population based study (*n* = 762) on the association of *FSHB* with FSH serum levels and sperm parameters. The 67 subjects, who were treated with FSH, showed a significant increase in sperm count, FSH and Inhibin B. T allele carriers additionally showed a significant increase in sperm concentration and total motile sperm count. The most impressive increase in sperm parameters was evident in the patients homozygous for T (compared to G allele carriers). Also the response rate in terms of doubling of sperm count was highest in the TT-group compared to GT and the wildtype GG carriers (*p* = 0.001). The authors point out the more severe increase in sperm count and quality in TT carriers compared to the increase seen in a general population of oligozoospermic men treated with FSH (54).

In the study by Simoni et al. the prime pharmacogenetic selection criterion was the *FSHR* p.N680S, whereby one group contained subjects homogenous for N and the other comprised homogenous S genotypes. Another inclusion criterion was a DFI>15% (Table 2). Total DFI decreased significantly in the homozygous N group after 3 months of treatment and in the consecutive follow-up visit after another 3 months (57.92−43.68%, *p* = 0.004) (64). These results are in contrast to the study by Selice et al. who found the homozygous S genotype of the FSHR to be the one benefiting more from FSH treatment by improving sperm concentration and total sperm number. Simoni et al. declare study-design and inter-laboratory variability in results of semen analyses as possible reasons for these divergent results (64).

Eventually *FSHB* c.-211G>T was also evaluated in this study; comparing the brighter DFI amongst the three different genotypes for *FSHB* –c.211G>T (GG/GT/TT) there was no significant difference amongst the groups. When *FSHR* and *FSHB* genotypes were combined, a significant improvement in sperm total—and brighter DFI was observed for the homozygous *FSHR* p.N680S N and homozygous *FSHB* G genotypes (*p* = 0.025) (64). This is in contrast to the study by Ferlin and colleagues, who found the homozygous wildtype of the *FSHB* c.-211G>T to respond the least on FSH treatment. Simoni argues with the “traffic light” model by Tüttelmann et al., where the combination *FSHR* c.2039A>G AA or AG and *FSHB* c.-211G>T GG are the carriers with the best combination for FSH action (Figure 3) (56, 64).

Pregnancy rates were reported in the studies by Simoni and by Selice. In the latter the rate was compared amongst couples with treated males vs. males without FSH therapy, but the differences (14.8% vs. 4.6%) were not significant (63) and female factors have not been considered. In the study by Simoni, twelve pregnancies were reported during the trial, 6 in each group, both after natural conception or ART (64).

Interestingly, in all studies there were no comparisons on primary endpoints between the study arms. The statistical analyses rather focused on longitudinal effects from baseline to the end of therapy within one distinct SNP-group, rather than comparing it to the results of another SNP-group. Therefore, the predictive value of SNPs in FSHR or FSHB or the combinations thereof on putative beneficial effects of FSH on spermatogenesis are almost impossible to conclude.

Taken together, at present several pharmacogenomics studies addressed the effect of FSH treatment on spermatogenesis in idiopathic infertile men. However, due to the fact that selection criteria, treatment phase, and endpoint definitions are varying, no reliable conclusions and consequences can be drawn in terms of clinical application.

## Considerations for Future FSH-based Pharmacogenetic Studies in Infertile Men

As of today the need for treatment options for infertile men are clearly documented, warranted and should be brought to the public, the reproductive societies and the centers which daily face these patients. Besides these recipients it is also of crucial importance to convince the pharmaceutical industry that curing male infertility is a worthwhile investment. The financial support by them is a backbone to conduct proper clinical trials, but at the same time a limiting step, since study designs do not always follow the most appropriate approach, but are influenced by economic views which clearly affect the solidity of studies.

Taken into account the increasing knowledge of the importance of FSH for qualitative and quantitative normal spermatogenesis, principles of FSH-based pharmacogenetic studies emerge, which we strive to outline in the following chapter.

At the experimental side definitely more studies on the already known SNPs affecting FSH action are needed. For example it now becomes clear that some of these SNPs are displaying gender specific differences. In the case of the c.-211G>T *FSHB* SNP the genotype (GT/TT) leads to a decrease of FSH serum levels in men, presumably by affecting the transcriptional activity. In women this SNP induces an upregulation of FSH serum levels (71, 72). Keeping in mind that the majority of functional studies is being conducted in female cells or unrelated cell lines such as HEK-293 cells, it becomes very clear that human Sertoli cell systems or suitable cell lines are strongly needed.

**Figure 4:**
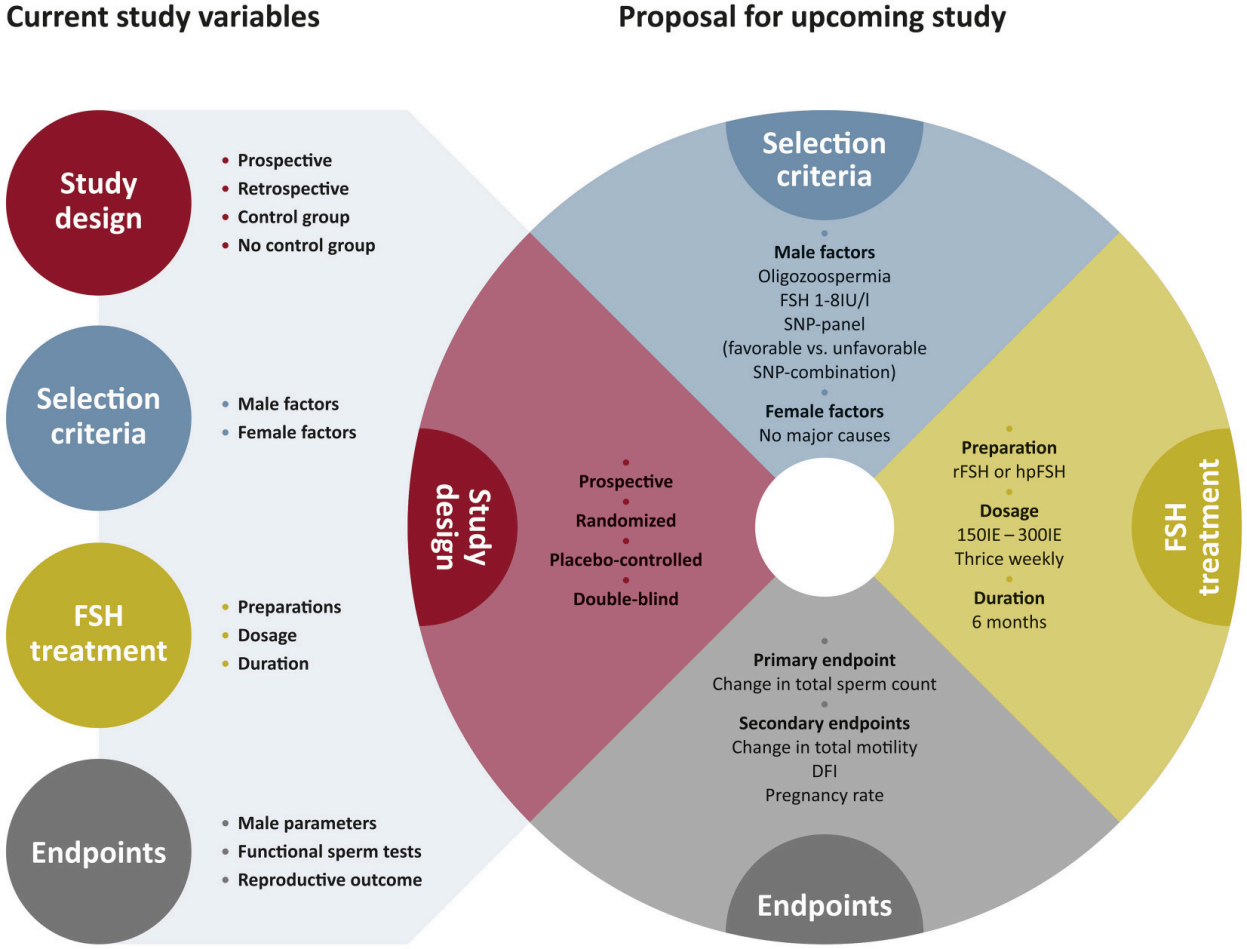
Current study outline and proposal for upcoming study outline. The current study outlines for pharmacogenetic studies are very heterogeneous and vary in many components. With the proposal for upcoming study outline we suggest to focus on the selected parameters to generate a substantial clinical study. The colored circles indicate the major critical components of a clinical study. The circles are complemented by the respective subgroups that contribute to this parameter. In the selected parameters for proposing upcoming study outlines, the colors of the artificial pie-chart correspond to the respective parameters like study design, selection criteria, FSH treatment, and endpoints on the left side.

At the genetic level it is to be assumed that the current work on SNPs from the *FSHB* and *FSHR* gene reflect only the tip of the iceberg and that there might be more upstream or downstream SNPs modulating FSH action. For example SNPs in transcription factors (e.g., SMAD, LHX3) might affect transcriptional regulation of *FSHB* or more downstream located SNPs in GDNF, or CXCL12 which impact FSHR signaling and thereby have an impact on spermatogenesis. Thus, it is crucial to decipher the FSH signaling network on spermatogenesis and to identify SNPs affecting this network. Using the potential of Genome Wide Association Studies (GWAS) this might provide novel insights into biological pathways, the discovery of novel target genes and the identification of SNPs influencing the FSH signaling network (73). It is to be envisaged that in the future a pharmacogenetic approach for identifying infertile men with a distinct set of unfavorable SNPs, will pinpoint individual patients eligible for FSH treatment and hence decrease the number of so far unexplained infertile males (idiopathic). However, the current outline of the studies is heterogeneous and shows a variety of study designs, treatments and endpoints as depicted in Figure 4. We therefore have made the attempt to propose an outline for FSH-based pharmacogenetic studies (Figure 4).

We suggest the study design to be prospective, randomized and placebo-controlled. Longitudinal evaluations within one SNP-group could reveal important information, but statistical analyses amongst the two study arms (FSH treatment vs. Placebo) are necessary, informative and can help to rule out individual variances.

For the selection of patients we suggest that two major criteria should be applied: the male and the female factor. If one wants to evaluate pregnancy rates after FSH treatment in the male, the female parameters are necessarily to be considered and mentioned. Concerning the male inclusion criteria we believe that rather commonly used sperm parameters like sperm count should be applied than functional sperm tests that reveal important additional information but are not part of a routinely used infertility workup. In terms of the pharmacogenetic selection of SNPs we strongly suggest to complement this by experimental studies (see above). By this translational approach the patient selection will be more precise, and the response to treatment putatively increased. There is no gold standard for the FSH treatment period and dosage, but a treatment for 6 months due to the spermatogenic cycle length of 74d, is most reasonable. From studies on patients with congenital hypogonadotropic hypogonadism we know that doses of 150 IU thrice weekly showed best treatment results, however these patients have another etiology of their impaired fertility and were treated with hCG as well (74). In a prospective, placebo-controlled clinical study in idiopathic infertile Chinese men, a FSH dose of 300 IU on alternate days for 5 months turned out to be successful (75). We therefore suggest that either dose can be applied. One of the major parameters for a successful study is the careful determination of endpoints. As suggested by the committee of the International Conference on Harmonization (ICH), on statistical principles the selection of the primary variable should reflect the accepted norms and standards in the relevant field of research (76). We therefore recommend taking the change in total sperm count as primary endpoint. Secondary endpoints may then be accomplished by further sperm parameters, functional sperm tests and pregnancy rate (after careful selection of female partners) (Figure 4).

Future trials could change the diagnostic and therapeutic work-up in patients with idiopathic infertility tremendously. Assessment of FSH polymorphisms and consecutive diagnose will be part of consulting and consequently will lead to FSH treatment options for a selected group of men. A significant increase of total sperm counts by FSH treatment could improve pregnancy rates, preferentially spontaneously conceived and thereby reduce the risks for the offspring caused by ART treatment.

## Author Contributions

MS, LP, and JG equally compiled the available literature, designed, and wrote the manuscript, and outlined the graphics.

### Conflict of Interest Statement

The authors declare that the research was conducted in the absence of any commercial or financial relationships that could be construed as a potential conflict of interest.
